# Functional Outcomes and Anti-Reflux Performance of Reconstruction Methods Following Proximal Gastrectomy: A Systematic Review

**DOI:** 10.3390/cancers18152476

**Published:** 2026-08-01

**Authors:** Kazuaki Tanabe, Ruxin Lei, Yoshihiro Saeki, Emi Chikuie, Hideki Ohdan

**Affiliations:** 1 Department of Perioperative and Critical Care Management, Graduate School of Biomedical and Health Sciences, Hiroshima University, 1-2-3 Kasumi, Minami-ku, Hiroshima 734-8551, Japan; d244803@hiroshima-u.ac.jp; 2 Department of Gastroenterological and Transplant Surgery, Graduate School of Biomedical and Health Sciences, Hiroshima University, 1-2-3 Kasumi, Minami-ku, Hiroshima 734-8551, Japan; iop890@hiroshima-u.ac.jp (Y.S.); chikuiee@hiroshima-u.ac.jp (E.C.); hohdan@hiroshima-u.ac.jp (H.O.); 3 Division of Endoscopic Surgery, Hofu Institute of Gastroenterology, Hiroshima University Hospital, 14-33 Ekiminami-machi, Hofu 747-0801, Japan

**Keywords:** proximal gastrectomy, reconstruction, esophagogastrostomy, double-tract reconstruction, double-flap technique, reflux esophagitis, quality of life, gastric cancer

## Abstract

Proximal gastrectomy preserves gastric function; however, reconstruction choice influences reflux control and quality of life (QOL), and the comparative efficacy of available techniques remains unclear. A PRISMA-guided search of PubMed and Web of Science Core Collection (January 2000–April 2025) identified studies reporting reflux or QOL outcomes after proximal gastrectomy. Eligible studies included adult gastric cancer cohorts with original clinical data. Two reviewers independently screened articles, extracted predefined variables, and performed a narrative synthesis. The protocol was archived on the OSF. Thirty-two studies (2958 patients) met inclusion criteria. Simple esophagogastrostomy (EG) consistently demonstrated the poorest reflux control and widest range of stricture rates. Double-tract reconstruction (DTR) showed a generally favorable balance among reflux control, low leakage rates, and acceptable stricture rates, suggesting that it may represent a practical and broadly applicable reconstruction option. Jejunal interposition and pouch variants were reported in small, heterogeneous series, limiting their generalizability. Among valve-forming methods, the double-flap technique was associated with very low rates of endoscopic reflux in two Los Angeles–graded cohorts, though a 4–6% stricture rate remained a concern. Evidence for the side overlap with fundoplication by Yamashita, both original and modified, was sparse and inconsistent. Global QOL, assessed using PGSAS-45/37 and EORTC instruments, did not differ consistently between reconstruction types, although specific domains—such as appetite loss, nausea, and weight maintenance—varied sporadically. Available evidence suggests that DTR may be considered a practical and broadly applicable reconstruction option following proximal gastrectomy, whereas the double-flap technique appears to provide strong reflux control but may be associated with a risk of anastomotic stricture. Simple EG may was associated with higher reflux rates across studies and may be less favorable for reflux control. Larger multicenter studies using standardized, nutrition-sensitive QOL measures and physiological reflux testing are needed to optimize reconstruction choice. However, substantial heterogeneity and the limited quality of the available evidence preclude definitive conclusions regarding the superiority of any single reconstruction method.

## Simple Summary

Proximal gastrectomy preserves part of the stomach and may support postoperative function, but the choice of reconstruction influences reflux and anastomotic outcomes. This systematic review included 32 studies involving 2958 patients and compared commonly used reconstruction methods. Simple esophagogastrostomy was technically straightforward but was associated with the highest reflux burden. Double-tract reconstruction generally provided a favorable balance between reflux control, leakage, and stricture, suggesting that it may be a practical option in many clinical settings. The double-flap technique showed particularly strong anti-reflux performance, although anastomotic stricture and technical complexity remain important considerations. Overall quality of life was broadly similar across reconstruction methods, but differences were observed in specific domains such as appetite, nausea, dysphagia, and weight maintenance. These findings clarify the relative strengths and trade-offs of each procedure and may support individualized reconstruction choice after proximal gastrectomy.

## 1. Introduction

Gastric cancer remains a leading cause of cancer-related mortality worldwide. According to WHO data from 2020 [1], gastric cancer accounts for 7.7% of cancer deaths, ranking fourth after lung (18.0%), colorectal (9.4%), and liver cancer (8.3%). This burden is particularly pronounced in East Asia, where both incidence and mortality rates are high. In Japan, early detection and appropriate treatment are crucial for improving patient outcomes. Recent advances in diagnostic imaging, endoscopy, and the biological characterization of tumors using biopsy speiemens have facilitated multidisciplinary treatment planning tailored to individual patients [2,3], leading to growing interest in surgical techniques that maintain postoperative quality of life (QOL) [4,5]. Surgical approaches that minimize complications and support long-term health are increasingly important. For upper-third gastric cancer, the main surgical options include total gastrectomy and proximal gastrectomy. Unlike total gastrectomy, Proximal gastrectomy removes only the proximal stomach but preserves the pyloric portion, thereby maintaining reservoir capacity and gastric endocrine function, and offering potential benefits for postoperative QOL. However, proximal gastrectomy has been infrequently selected due to high rates of reflux esophagitis and anastomotic stenosis, leading surgeons to prefer total gastrectomy for upper-third lesions [6,7]. Recently, several anti-reflux reconstruction methods have been developed, and Proximal gastrectomy has been re-evaluated. These approaches may help prevent reflux esophagitis, support weight maintenance, and reduce anemia [8,9]. In addition to conventional approaches such as simple esophagogastrostomy (EG), double-tract reconstruction (DTR), and jejunal interposition (JI), several new anti-reflux reconstruction techniques have emerged.

Previous systematic reviews and meta-analyses have compared the perioperative and functional outcomes of several reconstruction methods following proximal gastrectomy [10]. However, these analyses have generally focused on pooled comparisons between broadly classified reconstruction categories. Consequently, important differences among individual anti-reflux mechanisms, as well as the relationship between objective endoscopic reflux, patient-reported symptoms, and quality-of-life outcomes, remain insufficiently characterized. In addition, the evidence for recently developed techniques, including the double-flap technique and side-overlap esophagogastrostomy with fundoplication, is derived mainly from small and heterogeneous studies and has not been fully integrated into clinically oriented comparisons.

Therefore, this systematic review aimed to provide an updated and clinically focused synthesis of reconstruction methods following proximal gastrectomy. We separately evaluated objective endoscopic reflux, patient-reported reflux symptoms, anastomotic morbidity, and validated quality-of-life outcomes, while considering the specific anti-reflux mechanism of each procedure. By examining the balance between reflux prevention, anastomotic complications, and functional outcomes, this review sought to clarify the strengths and limitations of each reconstruction method and identify remaining priorities for future research.

## 2. Methods

### 2.1. Literature Search Strategy

This systematic review was conducted in accordance with the Preferred Reporting Items for Systematic Reviews and Meta-Analyses (PRISMA) 2020 guidelines [11]. The review protocol and data extraction templates are archived in the Open Science Framework for Transparency (https://doi.org/10.17605/OSF.IO/MPJF2, accessed on 30 July 2026). Database-specific search strategies were developed using the same search concepts related to gastric cancer, proximal gastrectomy, reconstruction methods, and postoperative functional outcomes. The complete search strategies for PubMed and the Web of Science Core Collection are provided in Supplementary Table S1. The PubMed search was last performed on 31 May 2025. The Web of Science Core Collection search was conducted on 22 July 2026. The search was restricted to English-language articles. Reference lists of included studies were manually screened to identify additional eligible publications.

### 2.2. Inclusion and Exclusion Criteria

Studies were included if they met all of the following criteria:

- Population consisted of adult patients (≥18 years) with histologically confirmed gastric cancer;
- Evaluated surgical outcomes following proximal gastrectomy, including reflux symptoms, nutritional status, and QOL.
- Original clinical research, including randomized controlled trials, non-randomized comparative studies, and prospective or retrospective observational studies.

The following exclusion criteria were applied:

- Studies involving pediatric patients;
- Animal or preclinical studies;
- Review articles, including systematic reviews and meta-analyses;
- Editorials, letters, or commentaries, or consensus statements;
- Study protocols;
- Scale-development studies;
- Single case reports;
- Studies without extractable clinical outcomes relevant to the review;
- Duplicate or overlapping publications that did not provide additional relevant data;
- Non-English-language publications.

#### Study Selection and Data Extraction

Two independent reviewers (K.T. and R.L.) screened titles and abstracts of retrieved articles, followed by full-text review of potentially eligible studies. Discrepancies were resolved through discussion until consensus was reached. For multiple publications from the same research group, only the most recent or comprehensive report was included to avoid duplication. From each included study, the following data were extracted using a standardized form:

- Study characteristics: first author, year of publication, journal
- Study design: randomized controlled trial, non-randomized comparative study, or prospective or retrospective observational study
- Patient characteristics: number of patients, age, tumor stage
- Surgical procedures: type of gastrectomy, reconstruction method
- Primary outcome 1—Reflux esophagitis: incidence and evaluation method (e.g., Los Angeles classification of reflux esophagitis)
- Primary outcome 2—QOL: assessment tool used (e.g., the Postgastrectomy Syndrome Assessment Scale-45 (PGSAS-45) and the European Organisation for Research and Treatment of Cancer Quality of Life Questionnaire-Stomach 22 (EORTC QLQ-STO22))
- Follow-up period: duration of postoperative follow-up (months or years).

### 2.3. Methodological Quality Assessment

The methodological quality of the included studies was assessed using the Joanna Briggs Institute (JBI) critical appraisal checklists appropriate to each study design. The JBI checklists for randomized controlled trials, cohort studies, analytical cross-sectional studies, and case series were applied as appropriate. Each item was rated as “yes,” “no,” “unclear,” or “not applicable.” Two reviewers independently assessed each study, and disagreements were resolved through discussion until consensus was reached. No numerical summary score or overall quality category was assigned; the appraisal was interpreted at the item level.

### 2.4. Data Synthesis

Owing to substantial clinical and methodological heterogeneity across the included studies, a quantitative meta-analysis was not considered appropriate. The studies differed in patient populations, study designs, surgical approaches, technical details of reconstruction, outcome definitions, assessment methods, and follow-up periods. In particular, reflux-related outcomes were evaluated using nonuniform measures, including patient-reported symptoms, endoscopic findings, medication use, and different grading systems. Technical variations were also present among studies evaluating the same reconstruction category. These differences limited the comparability of the reported data and precluded meaningful and reliable statistical pooling. Therefore, the findings were synthesized narratively according to reconstruction method and outcome domain and were summarized in tabular form.

## 3. Results

### 3.1. Study Selection

The database searches identified 74 records, including 66 from PubMed and 8 from the Web of Science Core Collection. After removing 9 duplicate records, 65 records remained for title and abstract screening. Of these, 29 records were excluded: 12 non-English-language publications, 11 non-original articles, 2 studies without relevant clinical outcomes, 1 retracted article, 1 consensus paper, 1 scale-development study, and 1 review article.

**Figure 1:**
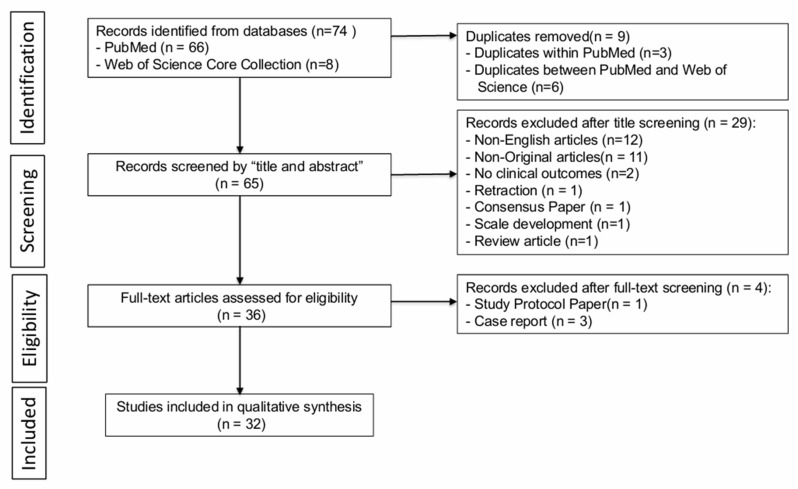
PRISMA 2020 flow diagram of study identification, screening, eligibility assessment, and inclusion based on searches of PubMed and the Web of Science Core Collection.

Thirty-six full-text articles were assessed for eligibility. Of these, 4 articles were excluded: 1 study protocol and 3 single-case reports. Finally, 32 studies were included in the qualitative synthesis. The study selection process is shown in Figure 1.

### 3.2. Study Characteristics

Thirty-two primary studies met eligibility criteria and formed the core of this review. Publication chronology was weighted toward the modern era: twenty-seven papers (84%) were published between 2015 and 2025, showing renewed interest in function-preserving gastrectomy, while five provided historical context from 1998. Geographically, the literature was almost exclusively East Asian—15 series from Japan, 11 from mainland China, 5 from the Republic of Korea, and 1 Canadian cohort, reflecting both the regional epidemiology of upper-third gastric cancer and surgical expertise. The study design was mainly observational, with twenty-three retrospective and six prospective cohorts; two randomized controlled trials and one quasi-experimental study provided higher-quality evidence. The studies analyzed 2958 patients who underwent proximal gastrectomy, with sample sizes from 16 to 301 (median = 64). Half of the studies enrolled only early stage disease (pathological stages I–II or cT1–T2N0), while others included mixed or unspecified stages, enhancing dataset external validity. Regarding the surgical approach, 17 studies involving 1300 patients included only minimally invasive procedures, including laparoscopic and robot-assisted surgery, whereas eight studies involving 800 patients included only open procedures. The remaining seven studies, comprising 858 patients, included both open and minimally invasive approaches, and in some of these studies, the number of patients undergoing each approach could not be determined separately. Primary reconstruction techniques fell into three categories—EG, DTR, and JI/JI with jejunal pouch interposition—listed by frequency. Several hybrid procedures have also been reported. Ten studies (31%) incorporated an anti-reflux mechanism—commonly fundoplication-based EG, the double-flap technique [8], or a modified EG method known as side overlap fundoplication by Yamashita [9]. The remaining studies relied on anatomical rearrangement. Follow-up reporting was adequate: twenty-four studies reported outcomes at ≥12 months (median follow-up, 24 months; range, 6–60), providing mature data for qualitative comparisons in Table 1 [12,13,14,15,16,17,18,19,20,21,22,23,24,25,26,27,28,29,30,31,32,33,34,35,36,37,38,39,40,41,42,43].

Available perioperative outcomes are summarized in Supplementary Table S2. Reporting was heterogeneous across studies, particularly with respect to the definitions and severity classifications of postoperative complications. Operative time was relatively frequently reported, whereas length of hospital stay and reoperation were reported less consistently. Intraoperative blood loss was sparsely reported and could not be meaningfully summarized. These differences precluded reliable direct comparisons among reconstruction methods.

### 3.3. Methodological Quality of the Included Studies

The methodological quality assessments are presented in Supplementary Table S3A–D. The two randomized controlled trials generally used comparable treatment groups and standardized outcome assessment; however, participant and surgeon blinding was not feasible, and outcome-assessor blinding was unclear. Among the cohort studies, outcome measurement and statistical analysis were generally appropriate, whereas adjustment for confounding factors and the reporting or management of incomplete follow-up were inconsistent. In the analytical cross-sectional studies, the principal concerns were insufficient strategies to address confounding and, in several studies, limitations in statistical analysis. The single case series had unclear reporting for several selection and setting-related items.

### 3.4. Reflux Esophagitis

Thirty of the 32 studies (2775 patients) reported at least one postoperative reflux outcome (Table 2) [12,13,14,15,16,17,18,19,21,22,23,24,25,26,27,28,29,30,31,32,33,34,35,36,37,39,40,41,42,43]. Nineteen studies assessed reflux endoscopically, with 16 using the Los Angeles (LA) classification [44]. Twenty-six studies reported symptom-based measures; most used the PGSAS-45/37 [45] and EORTC QLQ-STO22 [46] while others used Korean Quality of life in Stomach cancer patients Study group—40-item questionnaire (KOQUSS-40) [47] and GSRS [48] sporadically. Results are summarized by reconstruction type and compared based on presence of anti-reflux mechanism.

### 3.5. Simple EG

Thirteen studies (*n* = 720) evaluated EG without anti-reflux mechanisms. Endoscopic reflux was assessed in seven studies, five using the Los Angeles classification, with reflux rates from 8% to 27%. Symptom severity was evaluated using various instruments, mainly the PGSAS-45 (five studies, median score range: 2.0–2.6 on a 1–5 scale) and EORTC QLQ-STO22 (four studies, domain scores: 18–33/100). EG served as the comparator in nine head-to-head studies, mostly versus DTR. EG showed the highest or second-highest reflux burden among reconstruction types. Three studies reported statistically significant differences in favor of alternative methods, while others found no differences. These findings indicate EG is technically simple but potentially less favorable for reflux control. Anastomotic leakage after EG was reported in eight studies, ranging from 0% to 10.2%, mostly between 1% and 5%. Stricture was documented in six studies, with rates from 0% to 24.4%. One study showed a marginal increase in the EG group versus DTR (*p* = 0.068) [30], while others found no significant difference.

### 3.6. DTR

Thirteen studies (*n* = 831) evaluated the DTR after proximal gastrectomy. Nine studies assessed endoscopic reflux, seven using the LA classification, with reflux rates ranging from 5% to 18%. Five studies performed symptom-based evaluation—three using PGSAS-45/37 (median reflux subscale: 1.8–2.4 on a 1–5 scale) and two using EORTC QLQ-STO22 (reflux domain: 14–29/100). Other validated scales appeared sporadically. Three studies compared DTR with total gastrectomy followed by Roux-en-Y reconstruction; one showed higher reflux in DTR (*p* = 0.008), while two found no difference. Six studies compared DTR with simple EG [13,14,15,21,29,30]. Three showed DTR advantage, three found no differences. DTR consistently showed lower or comparable reflux burden relative to EG. Anastomotic leakage was reported in eight studies, rates 0% to 8.0%, mostly ≤4%. Strictures were reported in nine studies, rates 0% to 8.6%. Compared to EG, DTR showed similar or lower stricture incidence.

### 3.7. JI and Jejunal Pouch Interposition

Seven studies (*n* = 373) evaluated jejunal reconstruction after proximal gastrectomy. The cohort included 322 patients who underwent JI and 51 who underwent Jejunal pouch interposition. Endoscopic reflux was assessed in six studies, four using the LA classification; reflux rates ranged from 0% to 20.7%. Symptom assessment was reported in two studies—one using the PGSAS-45 (reflux subscale: 1.6–2.2 on a 1–5 scale) and one using the EORTC QLQ-STO22 (reflux domain: 11–26/100); no other validated instrument was used. A single comparison of JI versus simple EG showed no significant difference, though numbers favored JI. Two studies comparing JI and DTR reported equivalent reflux controls. One study that compared JI with Jejunal pouch interposition found no significant differences in reflux or complication rates. Anastomotic leakage was reported in three studies, with no leaks in patients who underwent either JI or Jejunal pouch interposition. Anastomotic stricture was documented in two studies, with rates ranging from 3.2% to 6.7% [12,34].

### 3.8. Impact of Anti-Reflux Reconstructions Including the Double-Flap Technique, Pseudofornix Technique, Side Overlap with Fundoplication by Yamashita, and Fundoplication-Augmented Esophagogastrostomy

Ten studies (*n* = 488) evaluated proximal gastrectomy reconstructions with anti-reflux mechanisms. Most patients underwent the double-flap technique (*n* = 221), followed by posterior or partial fundoplication-augmented EG (*n* = 147) and pseudofornix or side overlap with fundoplication variants (*n* = 88). Endoscopic reflux was reported in seven studies, six of which used the LA classification, with rates ranging from 0% to 25%. A review of the double-flap technique identified two LA-graded series (37 patients), with no cases of endoscopic reflux esophagitis (0%) [23,33]. No anastomotic leakage was reported, whereas stricture occurred in 5.0–5.6% of the double-flap technique cases, higher than the ≤2% reported for most other reconstructions. No studies reached statistical significance due to limited sample size. Symptom-based reflux assessment was performed in nine studies. The PGSAS-45/37 was used in six studies. Median reflux subscale scores ranged from 1.3 to 2.0 on a 1–5 scale and were lower than scores for simple EG arms [22,25].

### 3.9. Quality of Life

#### 3.9.1. Post-Gastrectomy Syndrome Assessment Scale (PGSAS-45/PGSAS-37)

Ten studies (*n* = 1050 cases of proximal gastrectomy) evaluated postoperative QOL using PGSAS instruments: six used the 45-item version and four used the 37-item abridged version [16,18,21,22,24,25,27,32,33,36]. Since symptom- and function-related domains were identical across versions, data were synthesized. Global QOL scores, including total symptom score and physical and mental component summaries, showed no significant differences among reconstruction methods. Intergroup means varied by less than 0.1 points, indicating no clinically meaningful differences. Two studies reported significant differences in weight maintenance subscales [21,24]. One found DTR associated with less postoperative weight loss than the group of gastric tube reconstruction, with no significant difference between DTR and simple EG [21]. Another study found shorter jejunal segments on DTR associated with better weight-related scores [24]. Anti-reflux-modified EG showed no improvement over simple EG in QOL outcomes [22,25,36]. For other subscales, including meal-related distress, dumping syndrome, and psychological dimensions, no consistent differences were observed. Lower dissatisfaction scores were reported for DTR in a single-center cohort, but these findings were not replicated and effect sizes were small [21]. The PGSAS-based data suggest current reconstruction methods offer comparable overall QOL after proximal gastrectomy, with modest, procedure-specific advantages in weight maintenance and patient dissatisfaction in selected cohorts.

#### 3.9.2. EORTC QLQ-C30/QLQ-STO22

Nine studies (*n* = 822 cases of proximal gastrectomy) evaluated postoperative QOL using the European Organisation for Research and Treatment of Cancer instruments, including the core QLQ-C30 and the gastric cancer–specific QLQ-STO22 modules [12,14,15,17,19,23,29,30,35]. These instruments assess global health status, functional domains, and symptom burden on a scale of 0–100, where higher scores indicate better functioning or greater symptom severity, depending on the domain. Global health status showed statistically significant intergroup differences in four studies. DTR yielded higher global scores than EG in two independent cohorts (83.3 vs. 50.0, both *p* < 0.001) [15,29]. In another study, JI outperformed both DTR and tube-like EG (83.3 vs. 75.0 vs. 70.8; JI vs. DTR, *p* = 0.021; JI vs. Tube-like stomach reconstruction, *p* < 0.001) [12]. A fourth study found superior global health scores for the Total Gastrectomy with Roux-en-Y reconstruction compared with those after the side overlap with fundoplication technique (83.3 vs. 75.0, *p* = 0.036) [17]. Other symptom domains showed procedure-specific differences in the isolated studies. DTR was associated with significantly lower nausea and appetite loss scores than EG in two studies (nausea: 25.0 vs. 66.7, *p* = 0.033; appetite loss: 33.3 vs. 66.7, *p* = 0.022) [15]; (nausea: 16.7 vs. 50.0, *p* < 0.001; appetite loss: 0.0 vs. 33.3, *p* < 0.001) [29]. In contrast, the DTR group showed worse outcomes than the double-flap technique group for dysphagia (17.7 vs. 5.9, *p* = 0.004) and eating restriction (24.8 vs. 15.2, *p* = 0.012) [23]. Anti-reflux EG using the side overlap technique demonstrated lower diarrhea scores (0.0 vs. 16.7, *p* = 0.012) and eating difficulty scores (4.2 vs. 12.5, *p* = 0.046) than the Total Gastrectomy with Roux-en-Y reconstruction [17]. Dysphagia severity also varied across the reconstruction groups; one study favored EG over DTR (11.1 vs. 0.0, *p* = 0.032), whereas another favored the double-flap technique [14,23]. In summary, while most symptom and functional domains assessed using the EORTC QLQ-C30/STO22 showed limited intergroup variation, certain subscales—such as dysphagia, appetite loss, nausea, diarrhea, and eating difficulty—revealed reconstruction-specific differences in isolated studies. However, these findings lack consistency across cohorts and should be interpreted with caution.

#### 3.9.3. Other QOL Instruments

In addition to the PGSAS and EORTC frameworks, eight studies employed alternative QOL assessment tools [13,28,31,37,39,41,43]. The Visick grading system, applied in two studies, suggested better symptom control with function-preserving procedures [28,31]. One of these studies also used the GSRS and reported lower total scores in the jejunal interposition group, indicating a better QOL. Another GSRS-based study found no overall differences, except for higher constipation scores in the EG group. The Spitzer QOL Index, used in one study, showed nutritional advantages but greater discomfort in the group that underwent anterior wall end-to-side anastomosis with pyloplasty, suggesting tradeoffs among reconstruction types [41]. The Functional Assessment of Cancer Therapy–Esophagus scale used in one study demonstrated improved social well-being after reconstruction [37]. Other instruments included the KOQUSS-40 (one study) and Likert-type institutional questionnaires (two studies); however, the results were inconsistent because of methodological variability [13,43].

## 4. Discussion

This systematic review qualitatively synthesized findings from 32 contemporary studies involving 2958 patients who underwent proximal gastrectomy followed by various reconstruction techniques. Four reconstruction types (EG, DTR, JI, and jejunal pouch interposition) were analyzed as the main representative methods, while anti-reflux procedures, such as the double-flap technique and the modified side overlap with fundoplication method, were evaluated. Other uncommon reconstructions have been described in the literature but were not systematically assessed in this review. Simple EG demonstrated the poorest reflux control, with LA–graded esophagitis reported in 8–27% of cases and anastomotic stricture in 0–24%. Across the included studies, DTR was generally associated with relatively low reflux rates, anastomotic leakage rates of up to 8%, and stricture rates of up to 8.6%. However, this technique involves two additional jejunal anastomoses, which may increase operative complexity and duration relative to EG-based procedures. These comparisons should be interpreted with caution given the heterogeneity of the study designs and patient populations. JI and jejunal pouch interposition were reported in smaller series, with reflux rates ranging from 0% to 20.7%, and no anastomotic leaks observed in the included cohorts. Strictures were reported in 3.2–9.3% of patients. Among valve-forming options, the double-flap technique was notable: in two LA-graded series totaling 37 patients, no endoscopic reflux was observed; PGSAS reflux subscale scores ranged from 1.8 to 2.6 on the 5-point scale, leakage ranged from 0% to 3%, and stricture, from 4% to 6%. However, these favorable findings were derived from only two small LA-graded cohorts totaling 37 patients and should therefore be interpreted cautiously. The limited sample size, small number of studies, and lack of adequately powered direct comparisons preclude firm conclusions regarding the superiority of the double-flap technique over other reconstruction methods. Symptom-based assessment was performed in nine studies, PGSAS-45/37 was used in six studies, and all comparisons favored the anti-reflux arm over the EG. Although endoscopic grading provides objective mucosal evidence, it only modestly correlates with patient-reported reflux. For example, in a post-gastric bypass cohort, reflux symptoms demonstrated high specificity (100%) but low sensitivity (41%) for detecting LA-graded esophagitis, underscoring the frequent mismatch between symptoms and endoscopic findings [49]. Our review demonstrated a similar mismatch across reconstruction types; procedures such as the double-flap technique or fundoplication-augmented EG were associated with very low rates of endoscopic reflux in the available studies, yet small residual scores persisted on the PGSAS or EORTC instruments. Conversely, several JI and jejunal pouch interposition studies have reported low symptom scores despite the presence of LA grade A–B esophagitis. Accordingly, future investigations should consider incorporating not only endoscopy and validated symptom and QOL instruments but also high-resolution impedance manometry [16] or 24-h pH-impedance monitoring [50] to better characterize postoperative reflux physiology. However, these advanced physiological assessments have thus far been limited to small exploratory series in the postgastrectomy setting, and long-term validation remains lacking. Beyond reflux-specific symptom scores, the evidence of broader differences in QOL remains limited. Nine studies employed the EORTC QLQ-C30/STO22 instruments [12,14,15,17,19,23,29,30,35] and four detected statistically significant intergroup differences in Global Health Status [12,15,17,29]. However, the preferred reconstruction method varied between the studies, and no single technique consistently outperformed the others (Table 3). Ten studies analyzed the composite PGSAS-45/37 score [16,18,21,22,24,25,27,32,33,35] and none found a statistically significant difference between the reconstruction types. Although individual subdomain analyses occasionally reported procedure-related differences—most often in appetite loss [15,29], nausea and vomiting [15,29], or weight maintenance [21,24]—these findings were inconsistent and did not establish a reproducible pattern. Interpretation of the QOL findings is limited by the use of different assessment tools and nonuniform follow-up periods across studies. Because the PGSAS and EORTC instruments evaluate partly different domains, and postoperative assessment times varied, the absence of consistent differences in global QOL scores should not be interpreted as evidence of equivalence among reconstruction methods. Long-term nutritional outcomes, including weight loss, anemia, vitamin B12 deficiency, and supplementation requirements, were also inconsistently reported. Moreover, most studies assessed postoperative outcomes within 2 years, precluding reliable evaluation and comparison of truly long-term nutritional consequences among reconstruction methods. Further studies are required to elucidate these domain-specific effects. However, evidence for side overlap with fundoplication-based reconstruction remains sparse and inconsistent. The original report of the side overlap with fundoplication by Yamashita (14 patients) reported no reflux [51], In contrast a prospective comparison with total gastrrectomy found LA grade B or higher esophagitis in 10.7% and overall reflux in 42.9% of 28 patients undergoing this procedure [17]. A separate 12-patient cohort undergoing the Y-shaped” modified side overlap with fundoplication reported a 17.8% reflux rate at one year [9]. All series reported zero anastomotic leakage or clinically significant strictures and noted favorable early quality-of-life outcomes or weight maintenance. Nevertheless, the small sample size and lack of randomized data indicate that the true anti-reflux efficacies of side overlap with fundoplication-based reconstructions remain uncertain and require multicenter validation.

Strengths of this review include its PRISMA-guided search strategy, 25-year literature window, and the separate synthesis of objective (endoscopy) and subjective (symptom and QOL) outcomes, allowing for clearer functional comparisons. These limitations stem primarily from the predominance of small retrospective studies with heterogeneous follow-up durations. Notably, nearly all the included studies originated in East Asia, where proximal gastrectomy is more frequently performed owing to differences in cancer screening programs and patient demographics. These regional factors, including disease epidemiology, body habitus, surgical training systems, and healthcare infrastructure, may limit the generalizability of our findings to non-Asian populations.

Clinically, DTR appears to provide a generally favorable balance between reflux control and postoperative safety and may represent a practical and broadly applicable reconstruction option, although its multi-anastomosis construction adds some operative complexity. The double-flap technique was associated with very low rates of endoscopic reflux in the available series, with stricture rates of 4–6%. These stricture rates may be numerically higher than those reported for some alternative techniques, although direct statistical comparisons are limited. The clinical significance of anastomotic stricture also depends on symptom severity, treatment requirements, and recurrence. Stricture may impair oral intake and adversely affect nutritional status and quality of life, while recurrent or refractory cases may require repeated endoscopic dilation. However, these aspects were inconsistently reported in the included studies, precluding reliable comparison of stricture severity and long-term impact among reconstruction methods. Surgeon experience, institutional expertise, and the procedural learning curve may also influence operative time and the risks of anastomotic leakage and stricture, particularly for technically demanding reconstructions such as the double-flap technique. Differences in institutional case volume, technical standardization, and perioperative management may therefore partly account for the variability observed across studies. However, these factors were inconsistently reported, precluding reliable assessment of their independent effects. Structured training and standardized operative techniques may be particularly important during the early adoption phase. Early robotic series indicated that robot-assisted double-flap technique may reduce reconstruction time and blood loss, potentially shortening the learning curve; however, the reported stricture rates have been variable, similar to or even higher than those of laparoscopic double-flap technique during the introductory phase [52]. These findings underscore the need for larger, high-quality, comparative studies evaluating robotic versus conventional laparoscopy.

Taken together, the current evidence suggests that DTR may represent a practical and broadly applicable reconstruction option, whereas the double-flap technique appears to provide strong reflux control but requires greater technical expertise and may carry a risk of anastomotic stricture. However, substantial heterogeneity and the limited quality of the available evidence preclude definitive conclusions regarding the superiority of any single reconstruction method. The variable results observed for side overlap with fundoplication-based redonstructions underscore how small single-center studies may obscure true procedural performance. Ultimately, these limitations highlight the need for next-generation reconstruction methods that combine the technical simplicity of EG with the anti-reflux efficacy of valve-forming techniques, an area of active innovation that warrants ongoing clinical evaluation.

## 5. Limitations

This review has several limitations. First, although the literature search was expanded to include both PubMed and the Web of Science Core Collection and was supplemented by manual screening of the reference lists of the included studies, other databases, including Embase, Scopus, and the Cochrane Library, were not searched. Therefore, the possibility that some relevant studies were missed cannot be completely excluded. However, the additional Web of Science Core Collection search identified no additional eligible studies. Second, we did not conduct a formal study-level risk of bias assessment for the included studies using standardized tools. Given that most studies were retrospective and single-center in nature, the findings may be affected by selection bias, confounding, and selective reporting, which should be considered when interpreting the results. Third, although the characteristics of the included studies were summarized in detail (Table 1), most of them were retrospective and heterogeneous in design, surgical approach, and outcome measures, which may introduce bias and limit the generalizability of the findings. Patient populations differed in terms of age, tumor stage, extent of gastric and esophageal resection, and perioperative management. Even within the same reconstruction category, technical details, such as the anastomotic configuration, length of the interposed jejunal limb, size of the gastric remnant, and operative modifications, were not uniform. Outcome definitions and assessment methods also varied considerably. In particular, reflux was assessed using heterogeneous measures, including patient-reported symptoms, endoscopic findings, medication use, and different grading systems. Follow-up periods and the timing and methods of nutritional and quality-of-life assessments were also inconsistent. These differences limit direct comparison of reported outcomes, may partly account for the variability across studies, and reduce the certainty and generalizability of the conclusions. The overall strength of the evidence is further limited by small sample sizes and variability in outcome definitions. Fourth, no quantitative meta-analysis was conducted because of substantial heterogeneity; therefore, results were synthesized narratively and presented in tabular form (Table 2 and Table 3). Fifth, no formal statistical exploration of heterogeneity, such as subgroup analyses or meta-regression, was performed. Potential heterogeneity across study design, patient population, surgical approach, and anti-reflux mechanisms was only described qualitatively. Sixth, because no quantitative meta-analysis was undertaken, sensitivity analyses to test the robustness of the synthesized results were not performed. Seventh, although a formal methodological quality assessment was performed, the evidence remained limited by the predominance of retrospective observational studies, incomplete control of confounding, and heterogeneity in outcome definitions and follow-up periods. Eighth, the certainty of the body of evidence was not assessed using a systematic approach such as GRADE, and the strength of recommendations is therefore limited. Nineth, No formal statistical assessment of publication bias was conducted because of the substantial heterogeneity among the included studies and the absence of a quantitative meta-analysis. In addition, selective outcome reporting cannot be excluded; favorable outcomes may have been preferentially published or reported, whereas complications, null findings, or unfavorable results may have been underreported. These potential biases should be considered when interpreting the conclusions. Finally, several methodological limitations of the review process should be noted. We did not perform a formal risk of bias assessment, quantitative synthesis, exploration of heterogeneity, sensitivity analyses, or certainty assessment, all of which may affect the robustness of the conclusions.

These methodological limitations should be considered when interpreting the findings of this review.

## 6. Conclusions

Proximal gastrectomy preserves gastric function but postoperative reflux remains an issue. This review shows that reconstruction choice primarily influences reflux control and anastomotic morbidity, while global QOL metrics show no consistent differences. However, domain-specific trends were observed, warranting further investigation. Simple EG shows higher reflux rates across studies and may be less favorable for reflux control. DTR appears to provide a generally favorable balance of reflux control, low leakage rates, and acceptable stricture incidence across the available studies and may represent a practical and broadly applicable reconstruction option. JI or pouch variants may be feasible, but the available evidence is limited to small, heterogeneous series and does not permit firm conclusions regarding their comparative effectiveness. Valve-forming techniques appear to provide strong anti-reflux effects in the available studies. The double-flap technique has been associated with very low rates of endoscopic reflux, although anastomotic stricture remains an important concern. Current evidence for side overlap with fundoplication-based reconstructions remains sparse and inconclusive. As the available literature is dominated by small, retrospective East Asian cohorts with variable follow-up durations, and high-quality randomized controlled trials remain scarce, adequately powered multicenter randomized trials using standardized definitions of reflux and nutritional outcomes, validated quality-of-life instruments, and objective physiologic reflux testing are needed to refine procedure selection and support evidence-based practice.

## Figures and Tables

**Table 1:** Summary of the characteristics and findings of the included studies.

NO	Authors (Year) [Ref. No.]	Study Period	Country	Study Design	Patients	Tumor Stage (Included)	Approach	Reconstruction Methods	Anti-Reflux Mechanism	Follow-Up Duration
1	Tian Z et al. (2025) [12]	2018–2022	China	Retrospective study	186	Stage not restricted	Laparoscopic Proximal Gastrectomy	JI/DTR/Tube-like stomach reconstruction	No	12 months
2	Oh S et al. (2025) [13]	2017–2019	Republic of Korea	Prospective observational study	49	Stage not restricted	Laparoscopic Proximal Gastrectomy/Robot-assisted Proximal Gastrectomy	DTR	No	12 months
3	Wang Z et al. (2024) [14]	2020–2021	China	Retrospective study	114	Stage not restricted	Laparoscopic Proximal Gastrectomy	DTR	No	12 months
4	Sun Y et al. (2024) [15]	2019–2023	China	Retrospective study	156	Stage not restricted	Laparoscopic Proximal Gastrectomy	DTR	No	12 months
5	Saeki Y et al. (2023) [16]	2015–2020	Japan	Retrospective study	16	pT1–T2 only	Laparoscopic Proximal Gastrectomy/Robot-assisted Proximal Gastrectomy	Double-flap technique	Yes	12 months
6	Zhang H et al. (2023) [17]	2019–2021	China	Prospective cohort study	21	cT1–T2N0 only	Laparoscopic Proximal Gastrectomy	Side overlap with fundoplication by Yamashita	Yes	12 months
7	Shibamoto J et al. (2023) [18]	2017–2021	Japan	Retrospective study	21	cT1–T2 only	Open Proximal Gastrectomy/Laparoscopic Proximal Gastrectomy/Robot-assisted Proximal Gastrectomy	Double-flap technique	Yes	6 months
8	Park J etal. (2023) [19]	2011–2015	Republic of Korea	RCT	68	cStage I only	Laparoscopic Proximal Gastrectomy	DTR	No	24 months
9	Gong J et al. (2023) [20]	2014–2019	China	Retrospective study	118	Stage not restricted	Open Proximal Gastrectomy	The uncut interposed jejunum pouch, esophagus and residual stomach double anastomosis/JI	No	24 months
10	Chen J et al. (2023) [21]	2017–2022	China	Retrospective study	164	Stage not restricted	Laparoscopic Proximal Gastrectomy	Gastric tube/DTR	No	12 months
11	Aizawa M et al. (2023) [22]	2018–2020	Japan	Prospective observational study	297	Stage not restricted	Open Proximal Gastrectomy/Laparoscopic Proximal Gastrectomy	EG/EG+ anti-reflux procedure	Yes	6 months
12	Yu B et al. (2022) [23]	2014–2018	China	Retrospective study	69	cStage I only	Laparoscopic Proximal Gastrectomy	DTR/Double-flap technique	Yes	12 months
13	Kamiya S et al. (2022) [24]	2018–2019	Japan	Cross-sectional study	172	Stage not restricted	Open Proximal Gastrectomy/Laparoscopic Proximal Gastrectomy	DTR	No	6 months
14	Aizawa M et al. (2022) [25]	2005–2020	Japan	Retrospective study	61	cT1N0 only	Open Proximal Gastrectomy/Laparoscopic Proximal Gastrectomy	EG	Yes	12 months
15	Toyomasu Y et al. (2021) [26]	2006–2017	Japan	Retrospective study	102	cT1–T2N0 only	Laparoscopic Proximal Gastrectomy	Gastric tube	No	>12 months
16	Sato R et al. (2021) [27]	2013–2019	Japan	Retrospective study	99	Stage not restricted	Laparoscopic Proximal Gastrectomy	DTR	No	24 months
17	Li Z et al. (2021) [28]	2007–2016	China	Retrospective study	301	Stage not restricted	Open Proximal Gastrectomy	JI/Gastric tube	No	3 months
18	JI X et al. (2021) [29]	2014–2019	China	Retrospective study	64	Stage not restricted	Open Proximal Gastrectomy/Laparoscopic Proximal Gastrectomy	DTR	No	12 months
19	Eom B et al. (2021) [30]	2013–2017	Republic of Korea	Retrospective study	103	cStage I only	Laparoscopic Proximal Gastrectomy	DTR	No	12 months
20	Yue C et al. (2020) [31]	2015–2017	China	Retrospective study	62	Stage not restricted	Open Proximal Gastrectomy	Antrum-preserving double tract gastric interposition reconstruction/JI	Yes	12 months
21	Yabusaki H et al. (2020) [32]	2009–2010	Japan	Prospective observational study	193	pStage I only	Open Proximal Gastrectomy/Laparoscopic Proximal Gastrectomy	EG/JI/JI with jejunal pouch interposition	No	12 months
22	Tsumura T et al. (2020) [33]	2011–2014	Japan	Retrospective study	19	pStage I only	Laparoscopic Proximal Gastrectomy	Double-flap technique	Yes	36 months
23	Nomura E et al. (2019) [34]	2012–2016	Japan	Prospective cohort study	30	cT1–T2 only	Laparoscopic Proximal Gastrectomy	DTR/JI	No	12 months
24	Park J et al. (2018) [35]	2011–2015	Republic of Korea	Retrospective study	34	Stage not restricted	Laparoscopic Proximal Gastrectomy	DTR	No	<24 months
25	Nishigori T et al. (2017) [36]	2006–2014	Japan	Retrospective study	20	cStage I only	Laparoscopic Proximal Gastrectomy	EG	Yes	12 months
26	Ronellenfitsch U et al. (2015) [37]	2005–2013	Canada	Retrospective study	50	Stage not restricted	Open Proximal Gastrectomy/Laparoscopic Proximal Gastrectomy	Gastric tube	No	6 months
27	Ohashi M et al. (2015) [38]	2007–2012	Japan	Prospective cohort study	65	cT1 only	Open Proximal Gastrectomy	JI	No	12 months
28	Ichikawa D et al. (2013) [39]	1997–2009	Japan	Retrospective study	39	pT1–T2 only	Open Proximal Gastrectomy	EG	Yes	8 months
29	Ahn S et al. (2013) [40]	2003–2009	Republic of Korea	Retrospective study	50	Stage not restricted	Laparoscopic Proximal Gastrectomy	EG	No	evaluated on symptoms
30	Zhang H et al. (2009) [41]	2002–2005	China	RCT	149	Stage not restricted	Open Proximal Gastrectomy	EG	No	6 and 24 months
31	Nakane Y et al. (2004) [42]	1993–2000	Japan	Retrospective study	35	pStage I–II only	Open Proximal Gastrectomy	JI/JI with pyloroplasty	No	24 months
32	Shiraishi N et al. (2002) [43]	1993–1999	Japan	Retrospective study	31	pStage I–II only	Open Proximal Gastrectomy	Gastric tube/JI	No	-

RCT: Randomized Controlled Trial Study, DTR: Double-tract reconstruction, EG: Esophagogastrostomy, JI: Jejunal Interposition.

**Table 2:** Summary of reflux.

No.	Author (Year)	Anti-Reflux Mechanism	Reconstruction Method (Number)	Anastomotic Leakage	Anastmotic Stricture	Reflux Evaluation Method	Result Regarding Reflux	Conclusion Regarding Reflux
1	Tian Z et al. (2025) [12]	No	JI (66) vs. DTR (62) vs. Tube-like stomach reconstruction (62)	0 (0) vs. 0 (0) vs. 1 (1.6%), *p* = 0.99	2 (3.2%) vs. 2 (3.2%) vs. 3 (4.8%), *p* = 0.862	Endoscopy	7 (11.3%) vs. 6 (9.7%) vs. 15 (24.2%), *p* = 0.046	Tube-like stomach reconstruction group demonstrated higher reflux
						EORTC QLQ-STO22-Reflux subscale	33.3 (22.2–66.7) vs. 33.3 (16.7–66.7) vs. 66.7 (47.2–75.0), *p* < 0.001	
2	Oh S et al. (2025) [13]	No	EG (18) vs. DTR (31)	Not reported	Not reported	KOQUSS-40-Reflux subscale	Δ = −8.48 (EG lower = worse), *p* = 0.067	No significant difference
3	Wang Z et al. (2024) [14]	No	DTR (87) vs. EG (96)	1 (1%) vs. 3 (2.6%), no significant difference	1 (1%) vs. 4 (3.5%), no significant difference	Endoscopy	8 (9.2%) vs. 26 (23.2), *p* = 0.009	DTR demonstrated fewer reflux
						EORTC QLQ-STO22-Reflux subscale	11.1 (0.22–22) vs. 22.2 (11.1–33.3), *p* = 0.001	
4	Sun Y etal. (2024) [15]	No	DTR (93) vs. EG (63)	4 (4.3%) vs. 5 (7.9%)	0 (0%) vs. 2 (3.2%)	EORTC QLQ-STO22-Reflux subscale	11.1 (5.55–27.8) vs. 48.9 (27.8–66.6), *p* = 0.030	DTR group demonstrated fewer reflux
5	Saeki Y et al. (2023) [16]	Yes	Double-flap technique (16) vs. the Japanese standard data of the PGSAS statics (Reflux)	Not reported	Not reported	PGSAS-37-Reflux subscale, HRIM	Reflux subscale: 2.0 ± 1.0 vs. 1.7 ± 1.0, *p* = 0.243	Some value in HRIM are correlated with reflux score
							The integrated relaxation pressure and Lower Esophageal Sphincter Residual Pressure measured (HRIM)correlated with Reflux subscale	
6	Zhang H et al. (2023) [17]	Yes	Side overlap with fundoplication by Yamashita (21) vs. Total Gastrectomy with Roux-en-Y reconstruction (34)	1 (3.6%) vs. 0 (0%), *p* = 0.999	0 (0%) vs. 0 (0%)	Endoscopy	Side overlap with fundoplication by Yamashita: 12 (42.9%)	Side overlap with fundoplication by Yamashita demonstrated higher reflux
						EORTC QLQ-STO22-Reflux subscale	22.2 (11.1–33.3) vs. 0 (0), *p* < 0.001	
7	Shibamoto J et al. (2023) [18]	Yes	Double-flap technique (21) vs. Total Gastrectomy with Roux-en-Y reconstruction (34)	Not reported	Not reported	PGSAS-37-Reflux subscale	Reflux subscale: 1.8 ± 0.8 vs. 2.0 ± 0.9, *p* = 0.541	No significant difference
8	Park J et al. (2023) [19]	No	DTR (68) vs. Total Gastrectomy with Roux-en-Y reconstruction (69)	Not reported	2 (2.9%) vs. 0 (0%), *p* = 0.25	Endoscopy (LA A–D),	2 (2.9%) vs. 2 (2.9%), *p* = 0.99	No significant difference
						EORTC QLQ-STO22-Reflux subscale	No significant difference	
10	Chen J et al. (2023) [21]	No	EG (51) vs. Gastric tube (77) vs. DTR (36)	2 (3.9%) vs. 0 (0%) vs. 0 (0%), *p* = 0.106	1 (2.0%) vs. 4 (5.2%) vs. 2 (5.6%), *p* = 0.615	Endoscopy (LA A–D)	19 (37.3%) vs. 18 (23.4%) vs. 5 (13.9%), *p* = 0.04	DTR group demonstrated fewer reflux
						PGSAS-45-Esophageal reflux subscale	Reflux subscale: 2.7 ± 1.4 vs. 2.7 ± 1.5 vs. 2.1 ± 1.5, *p* = 0.008 (DTR < EG) (*p* = 0.047, Cohen’s d 0.44) and Gastric tube (*p* = 0.046, Cohen’s d 0.041)	
11	Aizawa M et al. (2023) [22]	Yes	EG (21) vs. EG+anti-reflux procedure (264) (A: Double-flap technique (153), B: Pseudofornix and/or His angle (67) and C: Fundplication (44))	Not reported	Not reported	PGSAS-45-Esophageal reflux subscale	Reflux subscale: 2.6 ± 1.1 vs. 1.9 ± 1.0, *p* = 0.002, Cohen’s d 0.73	The group of anti-reflux procedure demonstrated fewer reflux.
							Reflux subscale of anti-reflux procedures: A: 1.8 ± 0.9 vs. B: 1.9 ± 1.1 vs. C: 2.1 ± 2.1, *p* = 0.217)	
12	Yu B et al. (2022) [23]	Yes	DTR (51) vs. Double-flap technique (18)	1 (2.0%) vs. 0 (0%)	1 (2.0%) vs. 1 (5.6%)	Endoscopy (LA A–D)	3 (5.9%) vs. 0 (0%), *p* > 0.999	No significant difference
						EORTC QLQ-STO22-Reflux subscale	15.8 vs. 11.1, *p* = 0.553	Double-flap technique group demonstrated a fewer tendency of taking anti-reflux medications
13	Kamiya S et al. (2022) [24]	No	DTR (172) (comparison by the size of remnant stomach, A: 1/3 (13), B: 1/2 (97), C: >2/3 (60), by the length of jejunum between esophagojejunostomy and jejunogastrostomy, D: <10 cm (62), E: >11 cm (97) and by the size of jenunogastrostomy, F: <5 cm, G: >6 cm)	Not reported	Not reported	PGSAS-45-Esophageal reflux subscale	A: 2.6 ± 1.2 vs. B: 2.0 ± 0.9 vs. C: 2.0 ± 1.0, *p* = 0.146	No significant difference in reflux regardless of remnant stomach size, length of interposed jejunum, or size of jejunogastrostomy
							D: 1.9 ± 1.0 vs. E: 2.2 ± 1.0, *p* = 0.171	
							F: 2.1 ± 0.9 vs. G: 2.0 ± 1.0, *p* = 0.683	
14	Aizawa M et al. (2022) [25]	Yes	EG (17) vs. EG with posterior fundoplication (44)	Not reported	Not reported	Endoscopy (LA A–D)	12 (70.6%) vs. 2 (0.5%), *p* < 0.01	EG with posterior fundoplication group demonstrated fewer reflux.
						PGSAS-45-Esophageal reflux subscale	Reflux subscale 2.4 ± 0.6 vs. 1.5 ± 0.5 (*p* < 0.01, Cohen’s d 1.75)	
15	Toyomasu Y et al. (2021) [26]	No	Gastric tube (102) vs. Total Gastrectomy with Roux-en-Y reconstruction (69)	3 (2.9%) vs. 1 (1.4%), *p* = 0.322	10 (9.8%) vs. 2 (2.9%), *p* = 0.008	Endoscopy (LA A–D)	17 (16.7%) vs. 7 (10.1%), *p* = 0.07	No significant difference
16	Sato R et al. (2021) [27]	No	DTR (99) vs. Total Gastrectomy with Roux-en-Y reconstruction (190)	5 (6.7%) vs. 1 (1.3%), *p* = 0.209	0 (0%) vs. 1 (1.3%), *p* = 1.000	Endoscopy (LA B-D)	6 (8.0%) vs. 0 (0%), *p* = 0.008	DTR group demonstrated higher reflux.
						PGSAS-45-Esophageal reflux subscale	1.97 vs. 1.89, *p* = 0.789 (12 months)	
17	Li Z et al. (2021) [28]	No	JI (150) vs. Gastric tube (151)	Not reported	Not reported	Endoscopy (LA A–D)	31 (20.7%) vs. 48 (31.8%), *p* = 0.028	JI group demonstrated fewer reflux.
18	JI X et al. (2021) [29]	No	EG (39) vs. DTR (25)	4 (10.2%) vs. 2 (8.0%)	Not reported	Endoscopy (LA A–D),	12 (30.8%) vs. 2 (8%), *p* = 0.032	DTR group demonstrated fewer reflux.
19	Eom B et al. (2021) [30]	No	EG (45) vs. DTR (58)	Not reported	11 (24.4%) vs. 5 (8.6%), *p* = 0.068	Endoscopy (LA A–D)	8 (17.8%) vs. 2 (3.4%), *p* = 0.041	DTR group demonstrated fewer reflux
						EORTC QLQ-STO22-Reflux subscale	11.1 (6.2–25.0) vs. 0 (0–19.4), *p* = 0.350	
20	Yue C et al. (2020) [31]	Yes	Antrum-preserving double tract gastric interposition reconstruction (32) vs. JI (30)	1 (3.1%) vs. 0 (0%), *p* = 0.329	Not reported	Endoscopy (LA B-D)	1 (3.33%) vs. 1 (3.13%), *p* = 0.467	No significant difference
21	Yabusaki H et al. (2020) [32]	No	EG (115) vs. JI (34) vs. Jejunal pouch interposition (44)	Not reported	Not reported	PGSAS-45-Esophageal reflux subscale	2.0 ± 1.0 vs. 2.1 ± 1.0 vs. 2.0 ± 0.9, *p* = 0.895	No significant difference
22	Tsumura T et al. (2020) [33]	Yes	Double-flap technique (19) vs. Total Gastrectomy with Roux-en-Y reconstruction (17)	0 (0%) vs. 1 (5%)	1 (5%) vs. 0 (0%)	Endoscopy (LA A–D)	0% vs. 6%	Double-flap technique demonstrated fewer esophagitis in endoscopy but no difference in PGSAS-45-Esophageal reflux subscale
						PGSAS-45-Esophageal reflux subscale	1.3 (1.1–2.5) vs. 1.5 (1.1–2.5), *p* = 0.3531	
23	Nomura E et al. (2019) [34]	No	DTR (15) vs. JI (15)	0 (0%) vs. 0 (0%)	1 (6.7%) vs. 1 (6.7%)	Endoscopy (LA A–D)	1 (6.7%) vs. 1 (6.7%)	No significant difference
24	Park J et al. (2018) [35]	No	DTR (34) vs. Total Gastrectomy with Roux-en-Y reconstruction (46)	Not reported	Not reported	EORTC QLQ-STO22-Reflux subscale	No significant difference, *p* = 0.458 (Graph only)	No significant difference
25	Nishigori T et al. (2017) [36]	Yes	EG (20) with posterior fundoplication vs. Total Gastrectomy with Roux-en-Y reconstruction (42)	1 (5%) vs. 1 (2%)	5 (25%) vs. 0 (0%), *p* = 0.002	Endoscopy (LA A–D)	5 (25%) vs. 3 (7%), *p* = 0.15	No significant difference
						PGSAS-45-Esophageal reflux subscale	1.5 (1–2.5) vs. 2.3 (1.5–2.8), *p* = 0.15	
26	Ronellenfitsch U et al. (2015) [37]	No	Gastric tube (50)	Not reported	Not reported	Endoscopy	7/24 (29.2%)	Gastric tube group demonstrated reflux in almost 30% of patients
						FACT-E-Heartburn subscale	Reflux symptoms: 10/30 (33.3%)	
28	Ichikawa D et al. (2013) [39]	Yes	EG with posterior fundoplication (39) vs. Total Gastrectomy with Roux-en-Y reconstruction (45)	0 (0%) vs. 0 (0%)	1 (2.6%) vs. 0 (%)	Endoscopy (LA A–D)	EG: 5/20 (25%) vs. Total Gastrectomy with Roux-en-Y reconstruction: Not reported	No significant difference
						GSRS-Reflux subscale	GSRS-Reflux: 1.9 vs. 2.1, *p* > 0.1	
						Frequency scale for the symptoms of GERD (more than 9 points)	15/32 (47%) vs. 25/40 (63%), *p* = 0.18	
29	Ahn S et al. (2013) [40]	No	EG (50) vs. Total Gastrectomy with Roux-en-Y reconstruction (81)	EG: 5 (10%) vs. Total Gastrectomy with Roux-en-Y reconstruction: Not reported	EG: 6 (12%) vs. Total Gastrectomy with Roux-en-Y reconstruction: 4 (4.9%)	Reflux symptoms according to Visick score	EG: 16 (32%) vs. Total Gastrectomy with Roux-en-Y reconstruction: 3 (3.7%), *p* < 0.001	EG group demonstrated higher incidence of reflux
			among EG: end-to-end anastomosis (EEA) (13) vs. side-to-side anastomosis (SSA) (37)	EEA: 1 (7.7%) vs. SSA: 4 (10.8%)	EEA: 6 (46.2%) vs. SSA: 0 (0%), *p* < 0.001	Reflux symptoms according to Visick score	EEA: 2 (15.4%) vs. SSA: 14 (37.8%), *p* = 0.135	
30	Zhang H et al. (2009) [41]	No	EG (149) Esophagogastric anterior wall end-to-side anastomosis combined with pyloroplasty (EA) (54) vs. Esophagogastric posterior end-to-side anastomosis (EP) (45) vs. Esophagogastric end-to-end anastomosis (EE) (50)	Not reported	Not reported	The Spitzer QOL index-Heart burden or belch subscale	6 months: EA: 1.37 ± 0.708 vs. EP: 0.73 ± 0.863, *p* < 0.005	EA group demonstrated significantly less heartburn or belching than EP group and EE group at 6 months after operation At 24 months as postoperative time progresses, results showed that heartburn or belching had improved, with EA group had significantly less heartburn or belching than EE group.
							EA: 1.37 ± 0.708 vs. EE: 0.80 ± 0.756, *p* < 0.005	
							EP: 0.73 ± 0.863 vs. EE: 0.80 ± 0.756, *p* = 0.547	
							24 months: EA: 1.78 ± 0.502 vs. EP: 1.73 ± 0.53, *p* = 0.651	
							EA: 1.78 ± 0.502 vs. EE: 1.38 ± 0.805, *p* = 0.005	
							EP: 1.38 ± 0.805 vs. EE: 1.38 ± 0.805, *p* = 0.023	
31	Nakane Y et al. (2004) [42]	No	JI (17) vs. JI with pyloroplasty (B) (18)	Not reported	Not reported	Endoscopy (LA A–D)	A: 0 (0%) vs. B 1 (6%)	No significant difference
32	Shiraishi N et al. (2002) [43]	No	Gastric tube (14) vs. JI (17)	Not reported	Not reported	Heartburn or belch subscale of the 22-item functional assessment scale proposed by Skeel	Gastric tube: 1.44 ± 0.53 vs. GI: 1.71 ± 0.61, *p* > 0.05	No significant difference

Studies No.9 and No.27 were excluded as they reported only clinical symptoms. DTR: Double-tract reconstruction, EG: Esophagogastrostomy, JI: Jejunal Interposition, EA: Esophagogastric anterior wall end-to-side anastomosis combined with pyloroplasty, EE: Esophagogastric end-to-end anastomosis, EP: Esophagogastric posterior end-to-side anastomosis. EORTC QLQ-STO: European Organization for Research and Treatment of Cancer Quality of Life Questionnaire—Stomach cancer–specific questionnaire, Endoscopy (LA A–D): Los Angeles (LA) classification of reflux esophagitis (Grades A–D), FACT-E: The Functional Assessment of Cancer Therapy—Esophageal Cancer subscale, GSRS: The gastrointestinal symptom rating scale, HRIM: High-resolution impedance manometry, KOQUSS-40: Korean Quality of life in Stomach cancer patients Study group—40-item questionnaire, PGSAS-45: The Post-Gastrectomy Syndrome Assessment Scale-45 questionnaire, PGSAS-37: The Post-Gastrectomy Syndrome Assessment Scale-37 questionnaires.

**Table 3:** Summary of QOL.

No.	Author (Year)	Anti-Reflux Mechanism	Reconstruction Method (Number)	QOL Assessment Tool	QOL Outcome	Conclusion (QOL)
1	Tian Z et al. (2025) [12]	No	JI (62) vs. DTR (62) vs. Tube-like stomach reconstruction (62)	EORTC QLQ-30	• Global health status: Single-tract jejunal interposition 83.3 > DTR 75.0 > Tube-like stomach reconstruction 70.8 (Single-tract jejunal interposition vs. DTR *p* = 0.021; Single-tract jejunal interposition vs. Tube-like stomach reconstruction *p* < 0.001). • Emotional functioning: Single-tract jejunal interposition 70.8 > DTR 66.7 > Tube-like stomach reconstruction 50.0 (Single-tract jejunal interposition vs. Tube-like stomach reconstruction *p* < 0.001).	Single-tract jejunal interposition group demonstrated a more favorable QOL
2	Oh S et al. (2025) [13]	No	EG (18) vs. DTR (31)	KOQUSS-40	• Global health score: mean Δ = −3.45 (EG lower = worse), *p* = 0.305	No significant difference
3	Wang Z et al. (2024) [14]	No	DTR (87) vs. EG (96)	EORTC QLQ-STO22	• Anxiety: 22.2 (0.33–33) vs. 33.33 (11.11–44.44), *p* = 0.008 • Dysphasia: 11.1 (1–11.1) vs. 0 (0–11.1), *p* = 0.032	DTR group demonstrated more favorable QOL
4	Sun Y et al. (2024) [15]	No	DTR (93) vs. EG (63)	EORTC QLQ-30, QLQ-STO22	• Global health status: DTR 83.3 > EG 50, *p* < 0.001 • Nausea and vomiting score: DTR 25 < EG 66.7, *p* = 0.033 • Appetite loss score: DTR 33.3 < EG 66.7, *p* = 0.022	DTR group demonstrated more favorable QOL
5	Saeki Y et al. (2023) [16]	Yes	Double-flap technique (16) vs. the Japanese standard data of the PGSAS statics (Reflux)	PGSAS-37	Lower Esophageal Sphincter Residual Pressure measured (HRIM) correlated with diarrhea score (*p* = 0.0412)	Lower Esophageal Sphincter Residual Pressure measured and the integrated relaxation pressure values correlated with reflux symptoms
6	Zhang H et al. (2023) [17]	Yes	Side overlap with fundoplication by Yamashita (21) vs. Total Gastrectomy with Roux-en-Y reconstruction (34)	EORTC QLQ-30, QLQ-STO22	• Global health status: Total Gastrectomy with Roux-en-Y reconstruction 83.3 > Side overlap with fundoplication by Yamashita 75, *p* = 0.036 • Diarrhea score: Side overlap with fundoplication by Yamashita 0 < Total Gastrectomy with Roux-en-Y reconstruction 16.7, *p* = 0.012 • Eating score: Side overlap with fundoplication by Yamashita 4.2 < Total Gastrectomy with Roux-en-Y reconstruction 12.5, *p* = 0.046 • Body weight loss at 1 year: Side overlap with fundoplication by Yamashita −10.5% < Total Gastrectomy with Roux-en-Y reconstruction −17.4%, *p* = 0.001	Side overlap with fundoplication by Yamashita demonstrated more favorable postoperative recovery, body weight, eating, and diarrhea.
7	Shibamoto J et al. (2023) [18]	Yes	Double-flap technique (21) vs. Total Gastrectomy with Roux-en-Y reconstruction (34)	PGSAS-37	• No significant difference in any of the QOL scores • Hemoglobin level at 1 and 6 months were higher in Double-flap technique group	No significant difference in QOL, but postoperative hemoglobin level is higher in Double-flap technique group
8	Park J et al. (2023) [19]	No	DTR (68) vs. Total Gastrectomy with Roux-en-Y reconstruction (69)	EORTC QLQ-30, QLQ-STO22	• No significant difference in Global health status • Physical functioning: DTR 89.5 > Total Gastrectomy with Roux-en-Y reconstruction 82.9 *p* = 0.03 • Social functioning: DTR 89.5 > Total Gastrectomy with Roux-en-Y reconstruction 82.4 *p* = 0.03 • Amount of Vitamin B12 supplementation (mg): DTR 0.4 (0–0) < Total Gastrectomy with Roux-en-Y reconstruction 2.5 (0–4)	DTR group demonstrated more favorable QOL
9	Gong J et al. (2023) [20]	No	The uncut interposed jejunum pouch, esophagus and residual stomach double anastomosis (43) vs. EG (36) vs. JI (39)	-	• PNI: The uncut interposed jejunum pouch, esophagus and residual stomach double anastomosis 50.5 ± 5.9 vs. EG 44.9 ± 4.5 vs. JI 47.8 ± 4.3, *p* < 0.0001 • Weight loss (kg): The uncut interposed jejunum pouch, esophagus and residual stomach double anastomosis 1.6 ± 0.8 vs. EG 3.9 ± 1.1 vs. JI 2.3 ± 1.3, *p* < 0.0001	The uncut interposed jejunum pouch, esophagus and residual stomach double anastomosis group demonstrated better postoperative nutritional status
10	Chen J et al. (2023) [21]	No	EG (51) vs. Gastric tube (77) vs. DTR (36)	PGSAS-45	• Change in body weight: −9.8 ± 8.6 vs. −12.6 ± 9.4 vs. −8.1 ± 5.5, *p* = 0.021 (Gastric tube > EG (*p* = 0.093, Cohen’s d 0.31) and DT (*p* = 0.002, Cohen’s d 0.55) • Dissatisfaction after meal: 2.8 ± 0.9 vs. 2.6 ± 1.0 vs. 2.3 ± 1.0, *p* = 0.031 (DT < EG (*p* = 0.009, Cohen’s d 0.58) and Gastric tube (*p* = 0.051, Cohen’s d 0.40) • Dissatisfaction for daily life subscale: 2.4 ± 0.6 vs. 2.4 ± 0.8 vs. 2.1 ± 0.7, *p* = 0.062) (DT < EG (*p* = 0.012, Cohen’s d 0.56) and Gastric tube (*p* = 0.064, Cohen’s d 0.38)	DTR group demonstrated more favorable QOL
11	Aizawa M et al. (2023) [22]	Yes	EG (21) vs. EG+anti-reflux procedure (264) (A: Double-flap technique (153), B: Pseudofornix and/or His angle (67) and C: Fundplication (44))	PGSAS-45	• Abdominal pain subscale: 2.2 ± 1.2 vs. 1.7 ± 0.8, *p* = 0.007, Cohen’s d 0.63 • Constipation subscale: 3.1 ± 1.3 vs. 2.5 ± 1.3, *p* = 0.0026, Cohen’s d 0.52	The group of anti-reflux procedure demonstrated fewer abdominal pain and constipation
12	Yu B et al. (2022) [23]	Yes	DTR (51) vs. Double-flap technique (18)	EORTC QLQ-30, QLQ-STO22	• Dysphagia score: DTR 17.7 > Double-flap technique 5.9, *p* = 0.004 • Eating restriction score: DTR 24.8 > Double-flap technique 15.2, *p* = 0.012 • Anxiety score: DTR 34.6 > Double-flap technique 20.9, *p* = 0.039 • Body image score: DTR 36.7 > Double-flap technique 17.7, *p* = 0.029	DTR group demonstrated more favorable QOL
13	Kamiya S et al. (2022) [24]	No	DTR (172) (comparison by the size of remnant stomach, A: 1/3 (13), B: 1/2 (97), C: >2/3 (60), by the length of jejunum between esophagojejunostomy and jejunogastrostomy, D: <10 cm (62), E: >11 cm (97) and by the size of jenunogastrostomy, F: <5 cm, G: >6 cm)	PGSAS-45	• Dissatisfaction with symptoms scale: A: 2.8 ± 1.1 vs. B: 2.0 ± 1.0 vs. C: 1.8 ± 0.9, *p* = 0.005 (A > C (*p* = 0.003, Cohen’s d 1.06) and A > B (*p* = 0.002, Cohen’s d 0.78)), D: 2.5 ± 1.1 vs. E: 2.2 ± 1.0 (*p* = 0.014, Cohen’s d 0.41), F: 2.4 ± 1.1 vs. G: 1.9 ± 0.9, (*p* = 0.022, Cohen’s d 0.49) • Dissatisfaction with meal scale: A: 3.2 ± 1.3 vs. B: 2.7 ± 1.2 vs. C: 2.4 ± 1.0, *p* = 0.035 (A > C (*p* = 0.043, Cohen’s d 0.8)), D: 2.5 ± 1.1 vs. E: 2.8 ± 1.2 (*p* = 0.082, Cohens’d 0.29), F: 3.1 ± 1.2 vs. G: 2.6 ± 1.1 (*p* = 0.0035, Cohen’s d 0.46) • Dissatisfaction with working scale: A: 2.9 ± 1.1 vs. B: 2.2 ± 0.9 vs. C: 1.7 ± 0.8, *p* < 0.001 (A > C (*p* < 0.001, Cohen’s d 1.45) and A > B (*p* < 0.005, Cohen’s d 0.87)), D: 1.7 ± 0.8 vs. E: 2.2 ± 1.1 (*p* = 0.003, Cohen’s d 0.49) • Dissatisfaction for daily life SS: A: 3.0 ± 1.0 vs. B: 2.2 ± 0.9 vs. C: 2.0 ± 0.7, *p* = 0.001 (A > B (*p* = 0.012, Cohen’s d 0.80) and A > C (*p* = 0.097, Cohen’s d 0.66), D: 2.0 ± 0.8, vs. E: 2.4 ± 0.9 (*p* = 0.005, Cohen’s d 0.47), F: 2.5 ± 0.9 vs. G: 2.1 ± 0.8 (*p* = 0.018, Cohen’s d 0.51) • Change in body weight score: D: −0.11 ± 0.08 vs. E: −0.14 ± 0.07 (*p* = 0.042, Cohen’s d 0.34) • Necessity for additional meals score: F: 2.6 ± 0.9 vs. G: 2.2 ± 0.9 (*p* = 0.05, Cohen’s d 0.42)	Larger remnant stomach, shorter length of interposed jejunum, and longer jejunogastrostomy demonstrated more favorable QOL
14	Aizawa M et al. (2022) [25]	Yes	EG (17) vs. EG with posterior fundoplication (44)	PGSAS-45	• No significant difference in any of the QOL scores	No significant difference in QOL
15	Toyomasu Y et al. (2021) [26]	No	Gastric tube (102) vs. Total Gastrectomy with Roux-en-Y reconstruction (69)	-	• postoperative bodyweight at more than 2.5 years: Gastric tube > Total Gastrectomy with Roux-en-Y reconstruction, *p* < 0.05 • postoperative hemoglobin at more than 1 year: Gastric tube > Total Gastrectomy with Roux-en-Y reconstruction, *p* < 0.05	Gastric tube group demonstrated more favorable nutritional status
16	Sato R et al. (2021) [27]	No	DTR (99) vs. Total Gastrectomy with Roux-en-Y reconstruction (190)	PGSAS-37	• No significant difference in any of the QOL scores • The percentage of body weight loss was lower in the DTR group but there was No significant difference.	No significant difference in QOL
17	Li Z et al. (2021) [28]	No	JI (150) vs. Gastric tube (151)	GSRS, Visick grade	• GSRS score: total 26.89 ± 5.06 vs. JI 25.58 ± 4.56 vs. Gastric tube 28.48 ± 5.78, *p* < 0.001 • Visick grade: lower in the JI, *p* < 0.05	JI group demonstrated more favorable QOL
18	JI X et al. (2021) [29]	No	EG (39) vs. DTR (25)	EORTC QLQ-30, QLQ-STO22	• Global health status: DT 83.3 > EG 50.0, *p* < 0.001 • Emotional functioning: DT 66.7 > EG 50.0, *p* < 0.001 • Nausea and vomiting: DTR 16.7 < EG 50.0, *p* < 0.001 • Appetite loss: DTR 0.0 < EG 33.3, *p* < 0.001 • Eating: DTR 25 < EG 41.7, *p* < 0.001 • Anxiety: DTR 55.6 < EG 41.7, *p* < 0.001	DTR group demonstrated more favorable nutritional status
19	Eom B et al. (2021) [30]	No	EG (45) vs. DTR (58)	EORTC QLQ-STO22	• No significant difference in any of the QOL scores	No significant difference in QOL
20	Yue C et al. (2020) [31]	Yes	Antrum-preserving double tract gastric interposition reconstruction (32) us JI (30)	Visick score	• Visick score: Antrum-preserving double tract gastric interposition reconstruction 1.53 ± 0.12 vs. ADJR 1.37 ± 0.14, no significant difference	No significant difference in QOL
21	Yabusaki H et al. (2020) [32]	No	EG (115) vs. JI (34) vs. Jejunal pouch interposition (44)	PGSAS-45	• Quality of ingestion subscale: EG 3.5 ± 1.0 vs. JI 4.0 ± 0.8 vs. Jejunal pouch interposition 3.5 ± 1.0, *p* = 0.022 JI > EG (*p* = 0.022, Cohen’s d 0.57), JI > Jejunal pouch interposition (*p* = 0.050, Cohen’s d 0.59) • Food-related distress subscale/Constipation subscale/Dumping subscale: Jejunal pouch interposition group tend to better than EG group • Dissatisfaction at working/Dissatisfaction for daily life subscale: Jejunal pouch interposition group tend to better than EG group	Several main QOL outcomes tend to better in Jejunal pouch interposition group than EG group
22	Tsumura T et al. (2020) [33]	Yes	Double-flap technique (19) vs. Total Gastrectomy with Roux-en-Y reconstruction (17)	PGSAS-45	• No significant difference in any of the QOL scores • Body weight loss (%): Double-flap technique 9.6% vs. Total Gastrectomy with Roux-en-Y reconstruction 15.1%, *p* = 0.0132	No significant difference in QOL, but Double-flap technique maintained postoperative body weight
23	Nomura E et al. (2019) [34]	No	DTR (15) vs. JI (15)	-	• No significant difference in body weight change	No significant difference in body weight change
24	Park J et al. (2018) [35]	No	DTR (34) vs. Total Gastrectomy with Roux-en-Y reconstruction (46)	EORTC QLQ-30, QLQ-STO22	• No significant difference in any of the QOL scores • Vitamin B12 injection required (mg): 1 (0–12) vs. 4 (0–12), *p* < 0.001	No significant difference in QOL, but DTR group demonstrated superior in preventing vitamin B12 deficiency
25	Nishigori T et al. (2017) [36]	Yes	EG (20) with posterior fundoplication vs. Total Gastrectomy with Roux-en-Y reconstruction (42)	PGSAS-45	• Diarrhea subscale: 1 (1.0–2.3) vs. 2.7 (2.0–3.0), *p* = 0.002 • Dissatisfaction with symptoms: 1 (1–2) vs. 2 (2–3), *p* = 0.002 • Body weight loss (%): 10.7% vs. 16.3%, *p* = 0.034	EG with posterior fundoplication demonstrated less postoperative body weight loss and better QOL than Total Gastrectomy with Roux-en-Y reconstruction despite higher rates of anastomotic stricture
26	Ronellenfitsch U et al. (2015) [37]	No	Gastric tube (50)	FACT-E	• FACT-E scale: 1–6 month 122.5 (97–142) vs. >6 months 147 (132–159), *p* = 0.003	Gastric tube group demonstrated a significant increase in QOL upon assessment more than 6 months after surgery compared to both the preoperative and early postoperative state.
27	Ohashi M et al. (2015) [38]	No	JI (65) vs. Total Gastrectomy with Roux-en-Y reconstruction (117)	-	• Number of dumping symptom: 7 (11%) vs. 35 (30%), *p* = 0.003 • Body weight loss (%): 12.5 ± 5.8 vs. 17.4 ± 6.4, *p* < 0.001 • Decline in serum hemoglobin (%): 7.0 ± 5.7 vs. 9.7 ± 5.4, *p* = 0.002	JI group demonstrated more favorable nutritional status
28	Ichikawa D et al. (2013) [39]	Yes	EG with posterior fundoplication (39) vs. Total Gastrectomy with Roux-en-Y reconstruction (45)	GSRS	• Constipation score: 2.5 vs. 1.8, *p* < 0.05	No differences in QOL, except that constipation scores were higher in the EG group.
29	Ahn S et al. (2013) [40]	No	EG (50) vs. Total Gastrectomy with Roux-en-Y reconstruction (81) among EG: end-to-end anastomosis (EEA) (13) vs. side-to-side anastomosis (SSA) (37)	-	• No significant difference in any of nutritional status	No significant difference in any of nutritional status
30	Zhang H et al. (2009) [41]	No	EG (149): Esophagogastric anterior wall end-to-side anastomosis combined with pyloroplasty (EA) (54) vs. esophagogastric posterior end-to-side anastomosis (EP) (45) vs. esophagogastric end-to-end anastomosis (EE) (50)	The Spitzer QOL index	6 months • Eating time: EA: 1.83 ± 0.80 > EP: 1.58 ± 0.38 (*p* = 0.005), EE: 1.42 ± 0.64 (*p* < 0.05) • Volume of food: EA: 1.2 ± 0.81 > EP: 0.60 ± 0.78 (*p* < 0.05), EE: 0.58 ± 0.70 (*p* < 0.05) 24 months • Body weight: EA: 1.48 ± 0.64 > EP: 1.13 ± 0.63 (*p* = 0.005), EE: 1.14 ± 0.05 (*p* = 0.03) • Postprandial discomfort: EA: 1.69 ± 0.61 > EP: 1.07 ± 0.92 (*p* < 0.05), EE: 1.22 ± 0.79 (*p* = 0.001)	EA group demonstrated more favorable QOL
31	Nakane Y et al. (2004) [42]	No	JI (17) vs. JI with pyloroplasty (B) (18)	-	• No significant difference in postoperative symptoms • Dietary intake: B > A (*p* < 0.05, Graph only, 12 months) • Change of body weight (%): B < A (*p* < 0.05 Graph only, 6 and 24 months) (*p* < 0.01 Graph only, 12 months)	JI with pyloroplasty group demonstrated more favorable nutritional status
32	Shiraishi N et al. (2002) [43]	No	Gastric tube (14) vs. JI (17)	the 22-item functional assessment scale proposed by Skeel	• Vomiting: Gastric tube: 1.11 ± 0.33 < JI: 1.57 ± 0.65, *p* < 0.05 • Performance status: Gastric tube: 1.33 ± 0.50 < 1.85 ± 0.54, *p* < 0.05	Gastric tube group demonstrated more favorable QOL

DTR: Double-tract reconstruction, EG: Esophagogastrostomy, JI: Jejunal Interposition, EA: Esophagogastric anterior wall end-to-side anastomosis combined with pyloroplasty, EE: Esophagogastric end-to-end anastomosis, EP: Esophagogastric posterior end-to-side anastomosis. EORTC QLQ-STO: European Organization for Research and Treatment of Cancer Quality of Life Questionnaire—Stomach cancer–specific questionnaire, FACT-E: The Functional Assessment of Cancer Therapy—Esophageal Cancer subscale, GSRS: The gastrointestinal symptom rating scale, HRIM: High-resolution impedance manometry, KOQUSS-40: Korean Quality of life in Stomach cancer patients Study group—40-item questionnaire, PGSAS-45: The Post-Gastrectomy Syndrome Assessment Scale-45 questionnaire, PGSAS-37: The Post-Gastrectomy Syndrome Assessment Scale-37 questionnaires, PNI: Prognostic Nutritional Index.

## Data Availability

All data generated or analyzed during this study are publicly available at the Open Science Framework (OSF): https://doi.org/10.17605/OSF.IO/MPJF2 (accessed on 30 July 2026).

## Supplementary Materials

The following supporting information can be downloaded at: https://www.mdpi.com/article/10.3390/cancers18152476/s1, Table S1: Database-specific search strategies. Table S2: Perioperative outcomes of the principal reconstruction methods following proximal gastrectomy. Table S3: (A) JBI critical appraisal of randomized controlled trials; (B) JBI critical appraisal of cohort studies; (C) JBI critical appraisal of analytical cross-sectional studies; (D) JBI critical appraisal of case series.
