# Supplementary material for: Functional Outcomes and Anti-Reflux Performance of Reconstruction Methods Following Proximal Gastrectomy: A Systematic Review
Source: Cancers (Basel). 2026 Aug 1;18(15):2476. doi: 10.3390/cancers18152476 (PMC13464839; doi:10.3390/cancers18152476)
Supplement: Supplementary file 1 [file cancers-18-02476-s001.zip › cancers-4430694-supplementary.pdf]

Supplementary Table S1. Database-specific search strategies

Database	Search date	Publication period	Search field	Search strategy	Records identified
PubMed	May 31, 2025	January 1, 2000— April 30, 2025	As specified in the search strategy	("Stomach Neoplasms"[MeSH] OR "Gastric Neoplasms" OR "Stomach Cancer") AND ("Proximal Gastrectomy") AND ("Anti-reflux reconstruction" OR "Esophagogastrostomy" OR "Double-tract reconstruction" OR "Jejunal interposition") AND ("Quality of Life"[MeSH] OR "Functional outcomes" OR "Postoperative outcome") AND (2000:2025[pdat])	66
Web of Science Core Collection	July 24, 2026	January 1, 2000— April 30, 2025*	Topic	("stomach neoplasm*" OR "gastric neoplasm*" OR "stomach cancer") AND "proximal gastrectomy" AND ("anti-reflux reconstruction" OR "esophagogastrostomy" OR "double-tract reconstruction" OR "jejunal interposition") AND ("quality of life" OR "functional outcomes" OR "postoperative outcome")	8

Abbreviations: MeSH, Medical Subject Headings

＊For the Web of Science Core Collection search, records published in 2025 were manually limited to those published through April 30, 2025.

Table S2. Perioperative outcomes of the principal reconstruction methods following proximal gastrectomy

NO	Authors [Ref. No.]	Reconstruction Methods	Operative time	Postoperative complications	Hospital stay(day)	Reoperation
1	Tian Z et al. [12]	JI(n=62)/DTR(n=62)/TLR(n=62)	JI:156.4±11.5min; DTR:151.8±12.1min; TLR:152.5±11.9min	JI: CD>IIIa, 9.7%; DTR: CD>IIIa, 6.5%; TLR: CD>IIIa, 9.7%	JI:7.4±3.1; DTR:7.3±2.9; TLR:7.7±4.1	NR
2	Oh S et al. [13]	DTR(n=49)	NR	NR	NR	NR
3	Wang Z et al. [14]	DTR(n=87)/EG(n=96)	DTR:230(210,255)min; EG:195(1180, 250)min	DTR:CD=3,2.2%; EG:CD=3,1%	DTR:7; EG:8	NR
4	Sun Y et al. [15]	DTR(n=93)/EG(n=63)	DTR:166.38min; EG:174.06min	DTR:CD III/IV,5.4%; EG:CD III/IV,7.9%	DTR:13.83; EG:13.11	NR
5	Saeki Y et al. [16]	DFT(n=25)	376.3min	CD=2,8.0%	NR	NR
6	Zhang H et al. [17]	SOFY(n=28)/TG (n=24)	SOFY:245±66.9min;TG:294±51.5min	SOFY:CD>1,3/28;TG:CD>1,4/24	SOFY:8(8-9.75); TG:8(8-9)	NR
7	Shibamoto J et al. [18]	DTR (n=21)/TG (n=34)	NR	DTR:CD≥II,5/21;TG:CD≥II,9/34	NR	NR
8	Park J etal. [19]	DTR (n=68)/ TG(n=69)	DTR:219.5±66.4min; TG:201.9±51.2min	NR	DTR:7.4±3.1; TG:7.7±4.1	DTR:1.5%; TG:2.9
9	Gong J et al. [20]	JI(n=39)/EG(n=36)	NR	JI:17.95%;EG:16.67%	NR	NR
10	Chen J et al. [21]	EG(n=51)/GT(n=77)/DTR(n=36)	EG:138.53min; GT:141.38min; DTR:163.06min	EG:CD≥IIIa, 2%; GT:CD≥IIIa, 6.5%; DTR:CD≥IIIA, 5.6%	EG:11.39; GT:10.83; DTR:11.72	NR
11	Aizawa M et al. [22]	EG(n=17)/EGF (n=449)	EG:150.5min; EGF:315.9min	NR	EG:12.4; EGF:10.6	NR
12	Yu B et al. [23]	DFT(n=18)/DTR (n=51)	DFT: 235.3min; DTR:228.6min	DFT:CD≥III, 5.6%; DTR:CD≥III, 3.9%	DFT:8.3; DTR:8.4	NR
13	Kamiya S et al. [24]	DTR(n=172)	NR	NR	NR	NR
14	Aizawa M et al. [25]	EG(n=300)	NR	NR	NR	NR
15	Toyomasu Y et al. [26]	GT(n=102)/ TG(n=69)	GT:199.4min; TG:214.0min	GT:CD≥III, 20.1%; TG:CD≥III, 9.9%	NR	NR
16	Sato R et al. [27]	DTR(n=75)/ TG(n=75)	DTR:312min; TG:311min	DTR:CD≥IIIa, 12%; TG:CD≥IIIa, 5.3%	DTR:9; TG:9	NR
17	Li Z et al. [28]	JI(n=150)/ GT(n=151)	JI:220min; GT:182min	NR	NR	NR
18	JI X et al. [29]	EG(n=39)/ DTR(n=25)	EG:195min; DTR:240min	CD=III, EG: 2.6%; DTR: 0%	EG:11; DTR:13	NR

Table S2. Continued

NO	Authors [Ref. No.]	Reconstruction Methods	Operative time	Postoperative complications	Hospital stay(day)	Reoperation
19	Eom B et al. [30]	EG(n=45)/ DTR(n=58)	NR	Severe anastomotic stricture:EG:11.1%; DTR:5.2%	NR	NR
20	Yue C et al. [31]	DTR(n=30)	142.6min	Anastomotic leakage:0%	10.9	NR
21	Yabusaki H et al.[32]	EG(n=115)/ JI(n=34)/ JPI(n=44)	NR	NR	NR	NR
22	Tsumura T et al. [33]	DFT(n=16)/ TG(n=13)	DFT: 280min; TG: 306min	Anastomotic leakage, DFT:0%; TG:5%, Anastomotic stricture, DFT:5%; TG:0%	DFT:12; TG:13	
23	Nomura Eet al.[34]	DTR(n=15)/ JIP(n=15)	DTR:352.5min; JIP:322.5min	Anastomotic leakage, DTR:0%; JIP:0% Anastomotic stricture, DTR:6.7%; JIP:6.7%	DTR:14.2 ; JIP:14.7	NR
24	Park J et al. [35]	DTR(n=34)/ TG(n=46)	DTR:240.7min; TG:212.9min	DTR:CD=II, 17.6%; TG:CD=II, 10.9%	DTR:8.7; TG:8.8	NR
25	Nishigori T et al. [36]	EG(n=20)/ TG(n=42)	EG:300min; TG:340min	CD≥III, EG:5%; TG:5% Stricture:EG:5%; TG:0%	NR	NR
26	Ronellenfitsch U et al. [37]	GT(n=50)	NR	CD≥I, 46%	NR	NR
27	Ohashi M et al. [38]	JI(n=65)/ TG(n=117)	NR	Stricture, JI:9%; TG:8%	NR	NR
28	Ichikawa D et al. [39]	EG(n=39)/ TG(n=45)	NR	NR	NR	NR
29	Ahn S et al. [40]	EG(n=50)/ TG(n=81)	EG:216.3min; TG:242.2min	Stenosis, EG:12%; TG:4.9%	NR	NR
30	Zhang Het al. [41]	EG(n=149)	NR	NR	NR	NR
31	Nakane Yet al. [42]	JI(n=35)	NR	NR	NR	NR
32	Shiraishi N et al. [43]	GT(n=14)/ JI(n=17)	GT:162.7min; JI:163.4min	NR	GT:18; JI:27.7	NR

Data are shown for the principal reconstruction groups; small or uncommon groups were omitted. Outcomes are presented as reported, except that means were calculated from individual values when necessary. Complications follow the original definitions and were not recalculated when patient and event counts were indistinguishable. NR, not reported.

Abbreviations: CD, Clavien–Dindo; DFT, double-flap technique; DTR, double-tract reconstruction; EG, esophagogastrostomy; EGF, EG with fundoplication; JI, jejunal interposition; JPI, jejunal pouch interposition; SOFY, side-overlap esophagogastrostomy by Yamashita; GT, gastric tube reconstruction; TLR, tube-like stomach reconstruction TG, total gastrectomy.

Supplementary Table S3A. JBI critical appraisal of randomized controlled trials

Assessment tool: Joanna Briggs Institute critical appraisal checklist. Source: https://jbi.global/sites/default/files/2020-08/Checklist_for_RCTs.pdf

No.	Study	Q1	Q2	Q3	Q4	Q5	Q6	Q7	Q8	Q9	Q10	Q11	Q12	Q13
8	Park J et al. (2023)	Y	Y	Y	N	N	U	Y	Y	Y	Y	Y	Y	Y
30	Zhang H et al. (2009)	U	U	Y	N	N	U	Y	U	U	Y	Y	Y	U

Item	JBI appraisal question	Coding
Q1	Was true randomization used for assignment of participants to treatment groups?	Y / N / U / NA
Q2	Was allocation to treatment groups concealed?	Y / N / U / NA
Q3	Were treatment groups similar at the baseline?	Y / N / U / NA
Q4	Were participants blind to treatment assignment?	Y / N / U / NA
Q5	Were those delivering treatment blind to treatment assignment?	Y / N / U / NA
Q6	Were outcomes assessors blind to treatment assignment?	Y / N / U / NA
Q7	Were treatment groups treated identically other than the intervention of interest?	Y / N / U / NA
Q8	Was follow up complete and, if not, were differences between groups in follow up adequately described and analyzed?	Y / N / U / NA
Q9	Were participants analyzed in the groups to which they were randomized?	Y / N / U / NA
Q10	Were outcomes measured in the same way for treatment groups?	Y / N / U / NA
Q11	Were outcomes measured in a reliable way?	Y / N / U / NA
Q12	Was appropriate statistical analysis used?	Y / N / U / NA
Q13	Was the trial design appropriate, and were deviations from the standard RCT design accounted for in conduct and analysis?	Y / N / U / NA

Notes: Methodological quality was assessed using the Joanna Briggs Institute (JBI) critical appraisal checklist appropriate to each study design. Assessments shown are the final consensus judgments of the reviewers. Y, yes; N, no; U, unclear; NA, not applicable.

Supplementary Table S3B. JBI critical appraisal of cohort studies

Assessment tool: Joanna Briggs Institute critical appraisal checklist. Source: https://jbi.global/sites/default/files/2020-08/Checklist_for_Cohort_Studies.pdf

No.	Study	Q1	Q2	Q3	Q4	Q5	Q6	Q7	Q8	Q9	Q10	Q11
1	Tian Z et al. (2025)	Y	Y	Y	Y	Y	Y	Y	Y	U	Y	Y
3	Wang Z et al. (2024)	Y	Y	Y	Y	N	Y	Y	Y	N	N	Y
4	Sun Y et al. (2024)	Y	Y	Y	Y	Y	Y	Y	Y	Y	NA	Y
6	Zhang H et al. (2023)	Y	Y	Y	Y	N	Y	Y	Y	Y	NA	Y
7	Shibamoto J et al. (2023)	U	Y	Y	Y	N	Y	Y	Y	U	U	Y
9	Gong J et al. (2023)	Y	Y	Y	Y	N	Y	Y	Y	U	U	Y
10	Chen J et al. (2023)	Y	Y	Y	Y	N	Y	Y	Y	U	U	Y
12	Yu B et al. (2022)	Y	Y	Y	Y	N	Y	Y	Y	Y	Y	Y
14	Aizawa M et al. (2022)	Y	Y	Y	Y	N	Y	Y	Y	Y	Y	Y
15	Toyomasu Y et al. (2021)	Y	Y	Y	Y	N	Y	Y	Y	U	U	Y
16	Sato R et al. (2021)	Y	Y	Y	Y	Y	Y	Y	Y	Y	Y	Y
17	Li Z et al. (2021)	Y	Y	Y	Y	N	Y	Y	Y	Y	Y	Y
18	Ji X et al. (2021)	Y	Y	Y	Y	Y	Y	Y	Y	U	U	Y
19	Eom B et al. (2021)	Y	Y	Y	Y	N	Y	Y	Y	U	N	Y
20	Yue C et al. (2020)	Y	Y	Y	Y	N	Y	Y	Y	Y	NA	Y
22	Tsumura T et al. (2020)	Y	Y	Y	Y	Y	Y	Y	Y	Y	N	Y
23	Nomura E et al. (2019)	Y	Y	Y	Y	N	Y	Y	Y	Y	NA	Y
24	Park J et al. (2018)	Y	Y	Y	Y	Y	Y	Y	Y	U	U	Y
25	Nishigori T et al. (2017)	Y	Y	Y	Y	Y	Y	Y	Y	Y	Y	Y
26	Ronellenfitsch U et al. (2015)	NA	NA	Y	Y	N	Y	Y	Y	Y	N	Y
27	Ohashi M et al. (2015)	Y	Y	Y	Y	N	Y	Y	Y	Y	N	Y
28	Ichikawa D et al. (2013)	Y	Y	Y	Y	N	Y	Y	Y	Y	N	N

No.	Study	Q1	Q2	Q3	Q4	Q5	Q6	Q7	Q8	Q9	Q10	Q11
29	Ahn S et al. (2013)	Y	Y	Y	Y	N	Y	Y	Y	U	N	Y
31	Nakane Y et al. (2004)	Y	Y	Y	Y	N	Y	Y	Y	U	N	Y
32	Shiraishi N et al. (2002)	Y	Y	Y	Y	N	Y	Y	Y	U	N	Y

Item	JBI appraisal question	Coding
Q1	Were the two groups similar and recruited from the same population?	Y / N / U / NA
Q2	Were the exposures measured similarly to assign people to exposed and unexposed groups?	Y / N / U / NA
Q3	Was the exposure measured in a valid and reliable way?	Y / N / U / NA
Q4	Were confounding factors identified?	Y / N / U / NA
Q5	Were strategies to deal with confounding factors stated?	Y / N / U / NA
Q6	Were the groups/participants free of the outcome at the start of the study (or at the moment of exposure)?	Y / N / U / NA
Q7	Were the outcomes measured in a valid and reliable way?	Y / N / U / NA
Q8	Was the follow-up time reported and sufficient to be long enough for outcomes to occur?	Y / N / U / NA
Q9	Was follow-up complete and, if not, were reasons for loss to follow-up described and explored?	Y / N / U / NA
Q10	Were strategies to address incomplete follow-up utilized?	Y / N / U / NA
Q11	Was appropriate statistical analysis used?	Y / N / U / NA

Notes: Methodological quality was assessed using the Joanna Briggs Institute (JBI) critical appraisal checklist appropriate to each study design. Assessments shown are the final consensus judgments of the reviewers. Y, yes; N, no; U, unclear; NA, not applicable.

Supplementary Table S3C. JBI critical appraisal of analytical cross-sectional studies

Assessment tool: Joanna Briggs Institute critical appraisal checklist. Source: https://jbi.global/sites/default/files/2020-08/Checklist_for_Analytical_Cross_Sectional_Studies.pdf

No.	Study	Q1	Q2	Q3	Q4	Q5	Q6	Q7	Q8
2	Oh S et al. (2025)	Y	Y	Y	Y	Y	N	Y	Y
11	Aizawa M et al. (2023)	Y	Y	Y	U	Y	N	Y	N
13	Kamiya S et al. (2022)	Y	Y	Y	Y	Y	Y	Y	N
21	Yabusaki H et al. (2020)	Y	Y	Y	Y	Y	N	Y	N

Item	JBI appraisal question	Coding
Q1	Were the criteria for inclusion in the sample clearly defined?	Y / N / U / NA
Q2	Were the study subjects and the setting described in detail?	Y / N / U / NA
Q3	Was the exposure measured in a valid and reliable way?	Y / N / U / NA
Q4	Were objective, standard criteria used for measurement of the condition?	Y / N / U / NA
Q5	Were confounding factors identified?	Y / N / U / NA
Q6	Were strategies to deal with confounding factors stated?	Y / N / U / NA
Q7	Were the outcomes measured in a valid and reliable way?	Y / N / U / NA
Q8	Was appropriate statistical analysis used?	Y / N / U / NA

Notes: Methodological quality was assessed using the Joanna Briggs Institute (JBI) critical appraisal checklist appropriate to each study design. Assessments shown are the final consensus judgments of the reviewers. Y, yes; N, no; U, unclear; NA, not applicable.

Supplementary Table S3D. JBI critical appraisal of case series

Assessment tool: Joanna Briggs Institute critical appraisal checklist. Source: https://jbi.global/sites/default/files/2020-07/Checklist_for_Case_Series_0.pdf

No.	Study	Q1	Q2	Q3	Q4	Q5	Q6	Q7	Q8	Q9	Q10
5	Saeki Y et al. (2023)	Y	Y	U	Y	U	Y	Y	Y	U	N

Item	JBI appraisal question	Coding
Q1	Were there clear criteria for inclusion in the case series?	Y / N / U / NA
Q2	Was the condition measured in a standard, reliable way for all participants included in the case series?	Y / N / U / NA
Q3	Were valid methods used for identification of the condition for all participants included in the case series?	Y / N / U / NA
Q4	Did the case series have consecutive inclusion of participants?	Y / N / U / NA
Q5	Did the case series have complete inclusion of participants?	Y / N / U / NA
Q6	Was there clear reporting of the demographics of the participants in the study?	Y / N / U / NA
Q7	Was there clear reporting of clinical information of the participants?	Y / N / U / NA
Q8	Were the outcomes or follow-up results of cases clearly reported?	Y / N / U / NA
Q9	Was there clear reporting of the presenting site(s)/clinic(s) demographic information?	Y / N / U / NA
Q10	Was statistical analysis appropriate?	Y / N / U / NA

Notes: Methodological quality was assessed using the Joanna Briggs Institute (JBI) critical appraisal checklist appropriate to each study design. Assessments shown are the final consensus judgments of the reviewers. Y, yes; N, no; U, unclear; NA, not applicable.
